# The Measurement of Little g: A Fertile Ground for Precision Measurement Science

**DOI:** 10.6028/jres.110.082

**Published:** 2005-12-01

**Authors:** James E. Faller

**Affiliations:** JILA, National Institute of Standards and Technology and the University of Colorado, Boulder, CO 80309-0440

**Keywords:** acceleration of gravity, encouraging creativity, Einstein, *g*, gravity, little *g*, measurement science, precision measurement

## Abstract

The occasion of the 100th anniversary of Einstein’s “golden year” provides a wonderful opportunity to discuss some aspects of gravity—gravitation being an interest of Einstein’s that occurred a few years after 1905. I’ll do this by talking about the measurement of little *g*, the free-fall acceleration on the Earth’s surface that is mainly due to the Earth’s gravity but whose value is also affected by centrifugal forces that are a result of the Earth’s rotation. I will also describe two equivalence experiments and a test of the inverse-square law of gravitation. Finally, I will make some observations on the science of precision measurement—a subject that underpins much of scientific progress.

## 1. Introduction

**Fig. 1:**
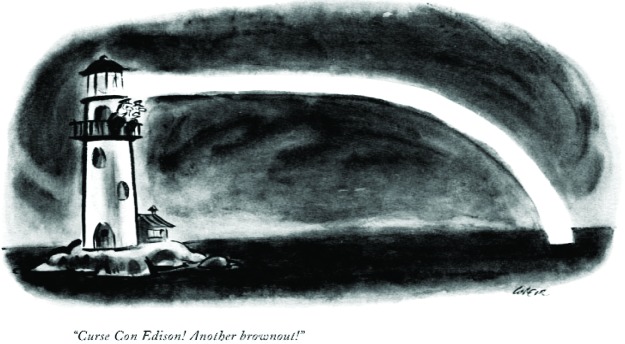
One effect of gravity. (© The New Yorker Collection 1973 Lee Lorenz from cartoon-bank.com. All Rights Reserved.)

**Fig. 2:**
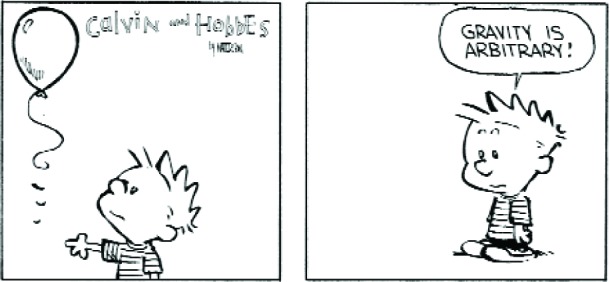
Gravity can be arbitrary. (Calvin and Hobbes © 1986 Watterson. Reprinted by permission of Universal Press Syndicate. All rights reserved.)

In discussing little *g*, I will focus on the fact that “apart from the fact that the basic physics of the (measurement) is easy to understand, it contains several examples of elegance and ingenuity” [1]. The Earth’s “gravity,” as is evidenced by little *g*, is known to everyone through familiar and dependable effects that touch nearly all aspects of life (Fig. 1). Yet, in the laboratory when trying to make instruments that measure little *g* work, gravity can sometimes seem rather arbitrary (Fig. 2).

One experiment that I will describe tested the equivalence of free fall for different masses in response to local attracting matter (the Earth) rather than to more distant matter (the Sun, which has been used in recent equivalence tests). The other equivalence test that I will mention was a result of the lunar laser-ranging experiment. The final measurement in this group of related experiments was a test of the inverse-square law of gravitation on the scale of meters to kilometers.

I also want to reflect on precision measurement—not, the reader needs to understand, precision measurements—by using these various measurements as examples of experiments of this type. Why not with the “s,” you might ask. The reason is that this area of physics is not just a collection of accurate measurements; rather it is an area of fundamental importance that involves measurement science applied to a broad range of *related* experiments and therefore no “s.”

**Fig. 3:**
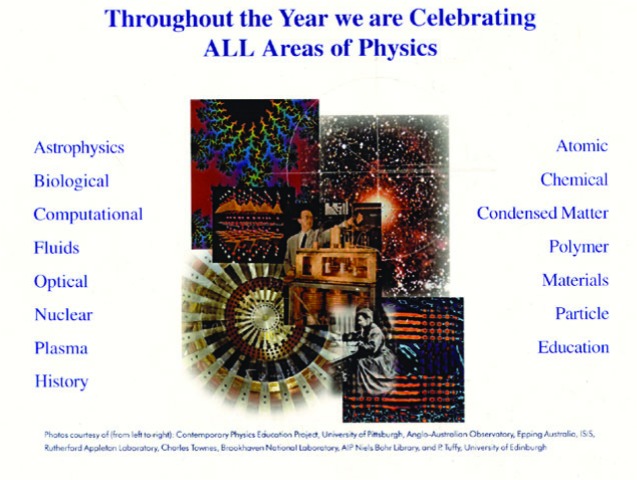
American Physical Society centennial year viewgraph. Used with permission of APS. (Photos courtesy of, from left to right, Contemporary Physics Education Project, University of Pittsburgh, Anglo-Australian Observatory, Epping Australia, ISIS, Rutherford Appleton Laboratory, Charles Townes, Brookhaven National Laboratory, AIP Niels Bohr Library, and P. Tuffy, University of Edinburgh.)

In spite of the importance of this field to scientific progress, precision measurement science is often overlooked. In March of 1999, when the American Physical Society had its centennial celebration, it provided a viewgraph (for the use of a group of centennial speakers—I was one) that was entitled “Throughout the Year we are Celebrating ALL (capitalization theirs) Areas of Physics” (Fig. 3). As you can see, neither gravitational physics nor precision measurement made the list of “ALL” areas of physics. When I enquired as to how these two areas “got lost,” I was told that there wasn’t room for them—despite the glaringly empty space following the last (important but rather arbitrary) entry, Education.

**Fig. 4:**
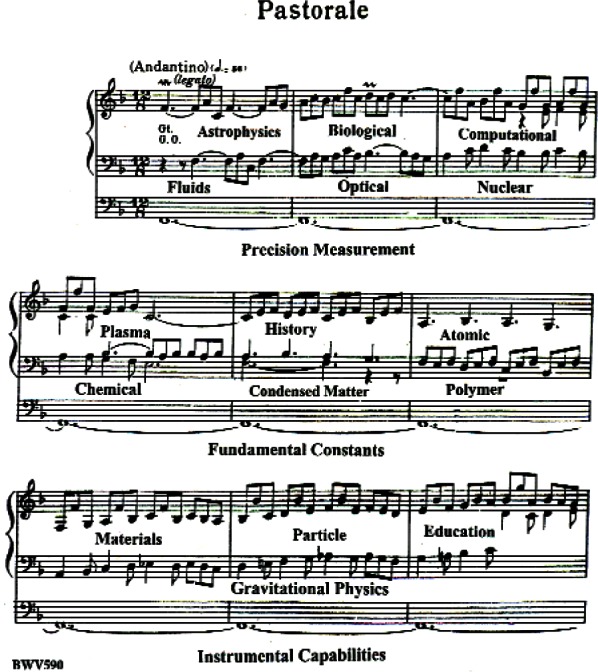
Embellished Pastorale in F (Johann Sebastian Bach).

This omission is difficult to understand because measurement capabilities are surely the enablers of almost all scientific progress. Indeed, instrumental capabilities, precision measurement, and fundamental constants all belong in the pedal line of the works we physicists discover and play for the world (Fig. 4). So, in keeping with Bach, I will try to point out the importance of these “pedal-line” scientific areas.

In this article, I will also grapple with the fascinating question of “where do ideas come from?” Since I was a student, I have always wondered how ideas came about. Particularly, during this special Einstein year, I find myself pondering what factors result in novel ideas … and if one isn’t an Einstein, whether one shouldn’t simply give up trying to come up with them.

At this point, vis-à-vis the question of how do ideas come about, let me relate an Einstein story that may give some insight into the workings of his mind. Some years ago, Einstein’s neighbor Professor Eric Rogers (author of *Physics for the Inquiring Mind* [2] and one of the great teacher/scientists that I have known) recounted to me this conversation “across a picket fence” that occurred in Princeton on Mercer Street in the early 1950s:

*Einstein:* How are things going?

*Rogers:* Fine. The only problem this semester is that for the hour between my 9 o’clock and 11 o’clock (physics for poets and pre-meds) classes, I literally have to lie down on the floor behind the lecture bench as my back is giving me a really hard time.

*Einstein:* Oh, let me give you the phone number of a doctor friend of mine from the old country who now practices in New York and is very good with back problems.

A few weeks later, a new conversation ensues across the same picket fence.

*Einstein:* Did you ever go and see my friend in New York?

*Rogers:* I did, and he helped me a lot. I no longer have to lie down between lectures with the result that I can use this hour to get something useful done. At his suggestion, I now wear a corset to help keep my back straight, and it really helps. It slips down from time-to-time requiring that I tug it back up, but that’s a small price to pay for the comfort it gives me.

Some five weeks later at 11 o’ clock at night, the phone rings in Eric’s study where he is working on his lecture for the following day. The caller says, “This is the professor.” (This is how Einstein introduced himself on the phone.)

*Einstein:* I’ve got it! (Eric had expected to hear the latest theory.) Suspenders!

Minds—apparently even Einstein’s—tend to “do science” subconsciously until, in the middle of something else, assorted bits of information coalesce into a solution for an existing problem or even a new idea. And “time off” from thinking about a specific problem I believe often helps the subconscious to provide a solution. In this regard, Rutherford punctually closed the Cavendish Laboratory at six o’clock each evening. His words were, “If one hadn’t accomplished what one wished to by 6 o’clock, it was unlikely that one would do so thereafter. It would be better to go home and think (I suspect he had in mind at least in part subconsciously while doing something else) about what one had done today and what one was going to do tomorrow [3].”

## 2. Getting Started

**Fig. 5:**
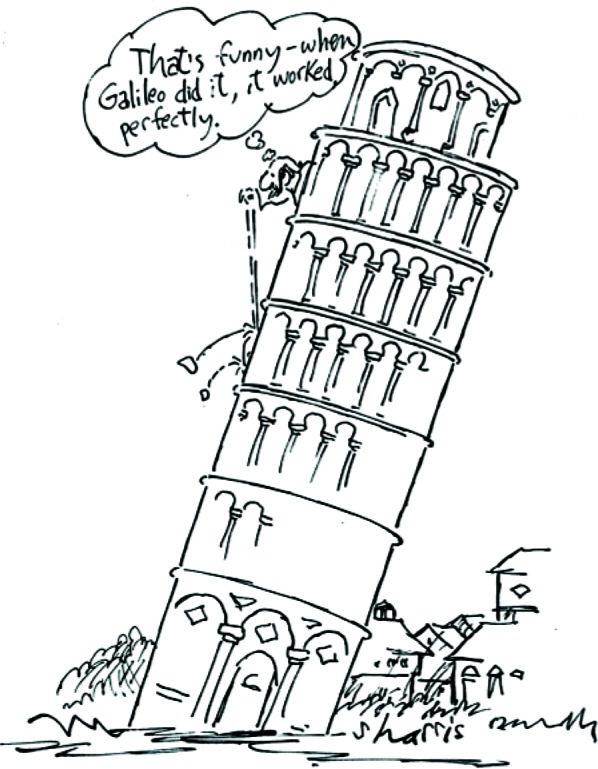
Practical guide to free-fall experiments. (Used with permission of ScienceCartoonsPlus.com.)

**Fig. 6:**
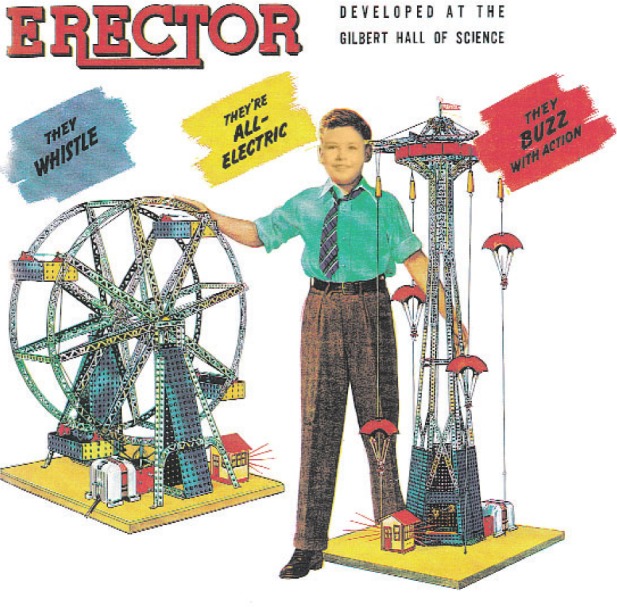
Erector Set. (Figure courtesy Eli Whitney Museum and the American Scientist.)

So now let’s begin by talking about how one might go about figuring out how to measure little *g*. A first instinct would be to rush to the library in hopes of finding a book that could be checked out and read along the lines of “A Practical Guide to Free-Fall Experiments” (Fig. 5). However, I believe that very little new science (almost by definition) is done that way. My “knowledge” of how to proceed, however, was almost certainly gained from my playing with the Gilbert 9-1/2 erector set 1(Fig. 6) that I had as a small boy—a Christmas gift from a spinster aunt. Incidentally, this hands-on “erector” experience is known to have strongly affected the careers of numerous scientists and engineers [4].

**Fig. 7:**
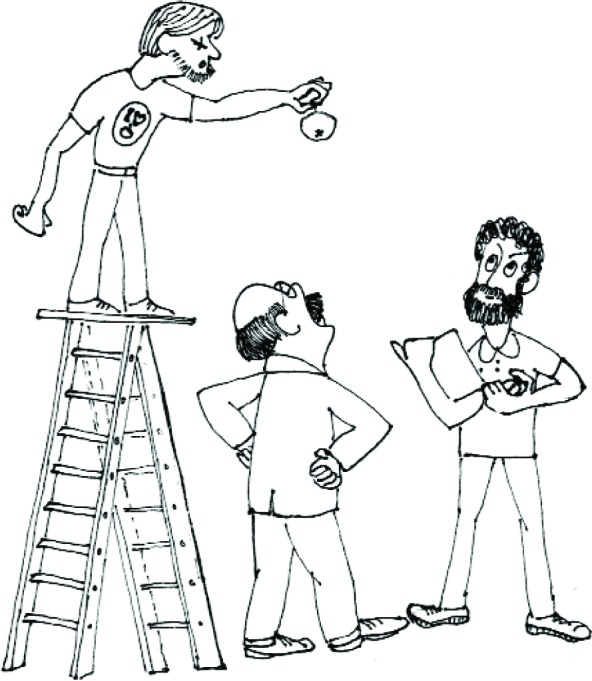
Theorist’s view of the measurement of *g*. (Used with permission of Zdenek Herman.)

**Fig. 8:**
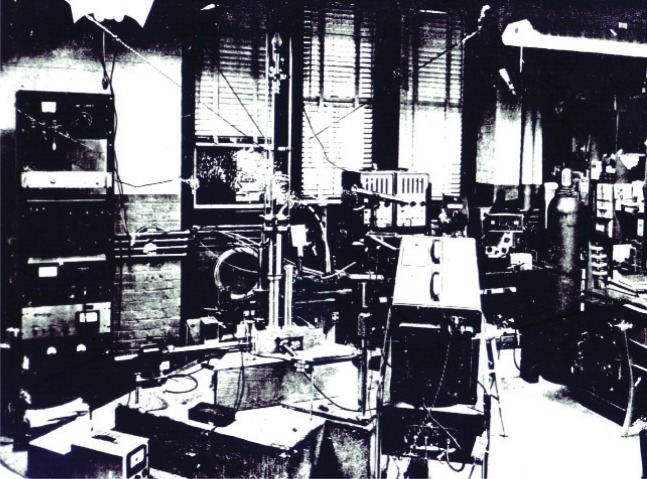
Laboratory scene from the late 1950s (lab photo).

In any event, today’s extraordinarily simple free-fall methods for measuring little *g* are schematically shown in Fig. 7. By the time I was a graduate student (1955), the pedal line—the technological means for carrying out such a straightforward approach to this measurement—existed: One could simply drop an object and measure its position, which increased quadratically with time, with sufficient precision to accurately determine *g*. Figure 8 shows how I got started dropping things [5–7]. It also shows much of the measurement capabilities of that day. There were no lasers. The scalars were capable of counting up to 1 MHZ. The oscilloscope was analog with a frequency response of 20 MHz. The ion pump’s technology was, however, nearly identical to that used in similar pumps today.

Lacking a laser, the brightest available light source for my gravity laboratory was the Sun. Outside the left-hand window was located a “Silvermann heliostat” that I used to steer the Sun into an optical interferometer arranged to produce three equally spaced white light fringes whose times of occurrence could be used to measure little *g*. During free fall, the characteristic time for the dropped object to move one fringe is on the order of 0.1 µs—much too short to “see” with the number of photons available from spectroscopic light sources. Standard (monochromatic) lamps were then employed in a separate measurement to determine the spacing of the three white light fringes.

**Fig. 9:**
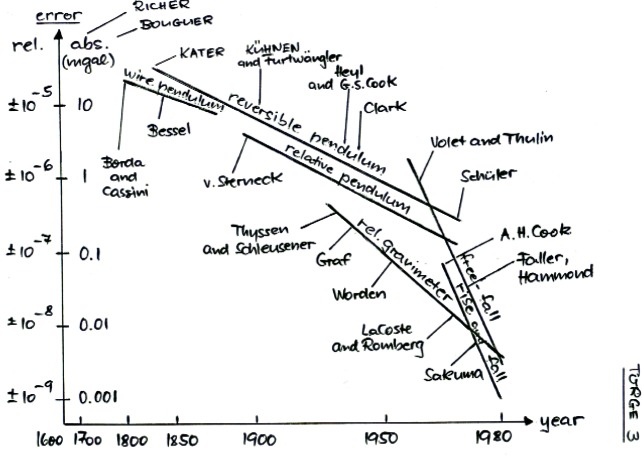
Progress in measuring *g* between 1680 and 1980. (Figure courtesy W. Torge, University of Hannover and Institüt für Erdmessung.)

The graph in Fig. 9 (given to me by Wolfgange Torge of the University of Hannover) shows the progress in measuring *g* during the period from 1680 to 1980. It begins on the far left with wire pendulums that were soon followed by absolute, knife-edge-supported, reversible pendulums. Following this, advances included the (a-stable) relative spring gravimeters, free-fall measurements made by dropping macroscopic objects and using the methods of geometrical optics to sense motion, and finally, free-fall measurements made using optical interferometry. These various advances led to over four orders of magnitude improvement in the measurement accuracy of little *g* over this 300 year period.

**Fig. 10:**
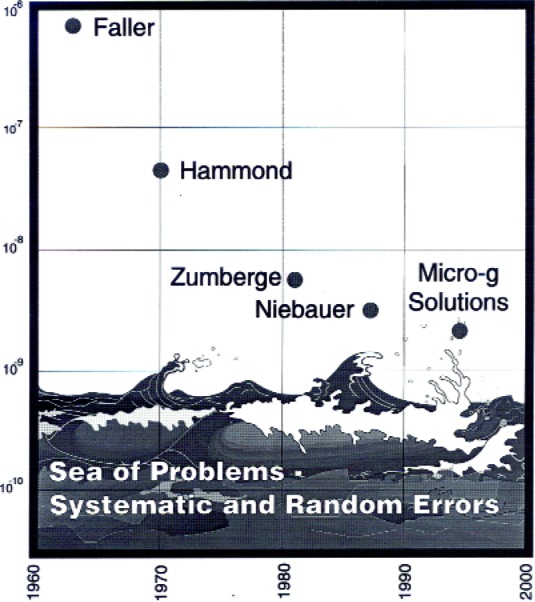
Measurement uncertainty in the absolute measurement of *g* versus time.

Recently, the measurement accuracy has improved even faster with time. Figure 10 shows the decreasing uncertainty in little *g* measurements performed in the United States from 1960 through 2000—nearly three orders of magnitude improvement during this 40-year time frame. This progress is a tribute to both clever ideas and technological advances. In this fairly narrow field, as I believe it does in others, each generation of instruments stands on the twin shoulders of newly developed technology and ideas developed by the preceding generation.

**Fig. 11:**
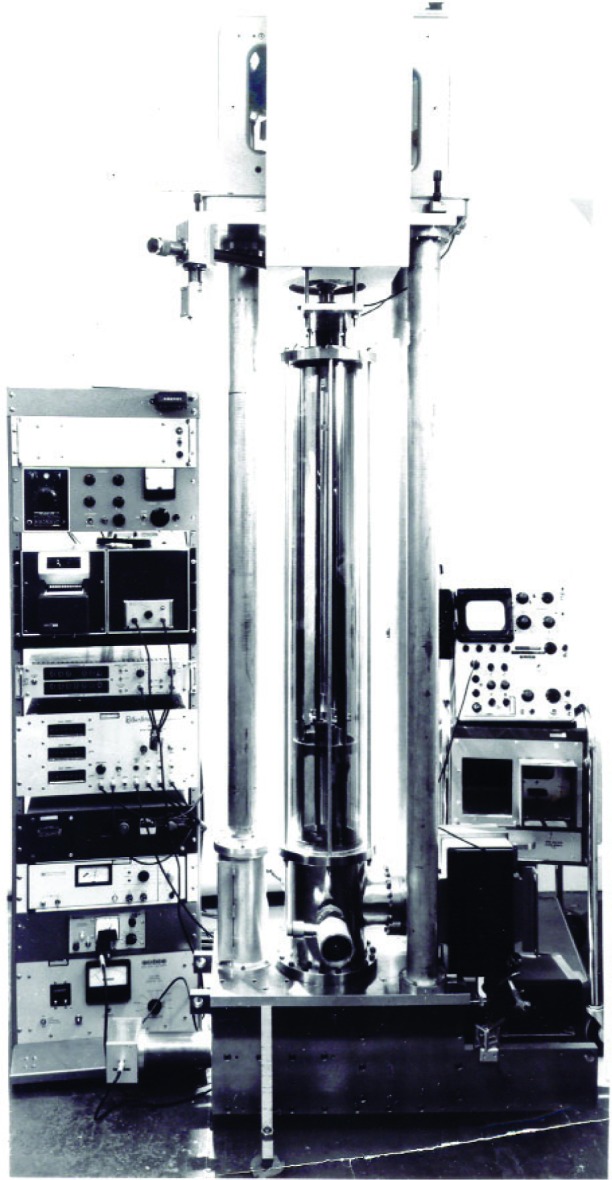
Laser interferometer absolute gravimeter (lab photo).

**Fig. 12:**
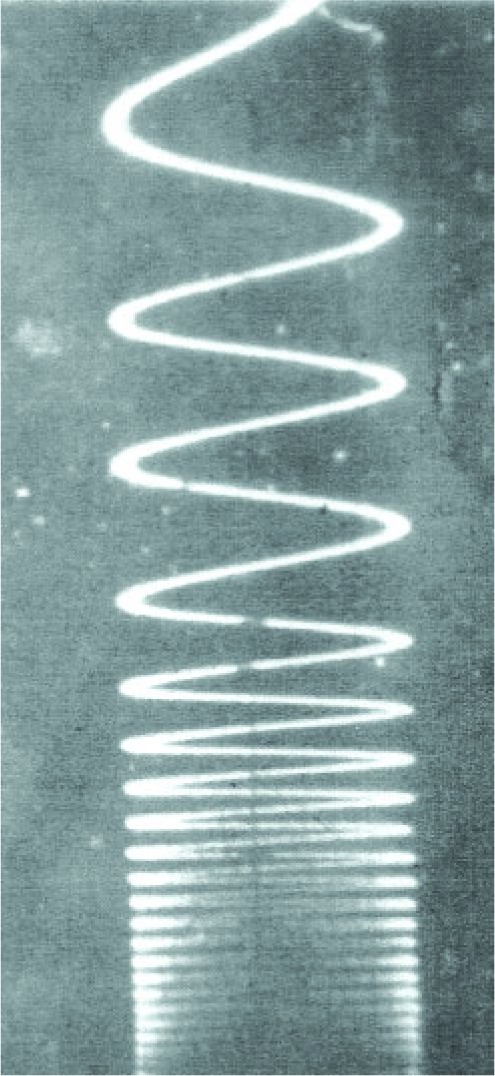
Fringe signal on dropping one of the two mirrors in an interferometer.

What were some of these advances? First and foremost, there was the laser, which provided both the brightness and the optical coherence to allow interferometry over meter-scale path lengths. Figure 11 shows the first of the laser interferometric absolute gravimeters, which was developed as part of the Ph.D. thesis of James Hammond [8]. It used a 5 parts in 10^8^ Spectra-Physics Lamb-dip stabilized laser—the most stable laser of its day, a free-fall length of about a meter, and a modified commercial long-period seismograph (where the reference “mirror” was attached to the seismometer’s cantilevered mass) to isolate the reference mirror from vibrations. The freely falling mirror was isolated by being in inertial space; the long-period isolator served to also locate the reference mirror in inertial space. Figure 12 shows the fringe trace that results when one mirror of a Michelson-type interferometer is allowed to free fall.

A frequency range of a few Hz to ≈10 MHz results during a free fall of ≈0.5 m. To determine *g*, “all” that was needed was to electronically note the time of passage of a subset of the resulting fringes [9]. Incidentally, the instrument shown in Fig. 11 was used to make the first transatlantic absolute measurements (at Middletown, Connecticut; Teddington, England; and Sévre, France) [10].

**Fig. 13:**
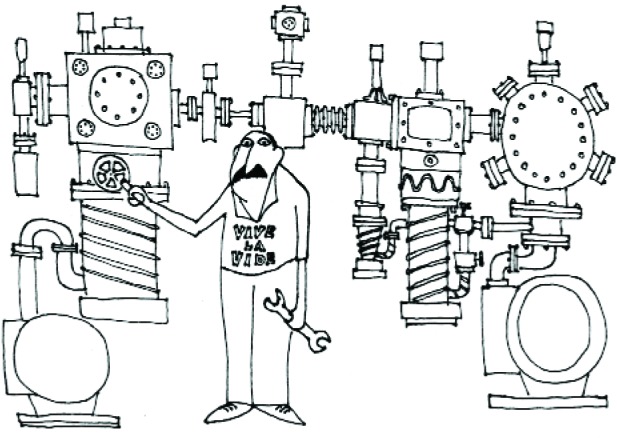
The vacuum engineer. (Used with permission of Zdenek Herman.)

**Fig. 14:**
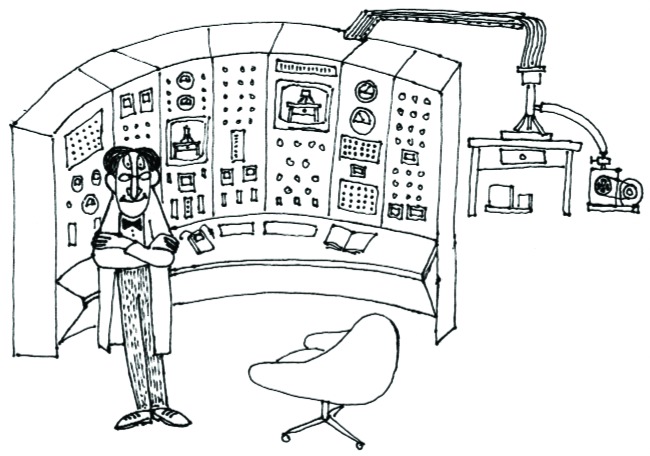
The computer meister. (Used with permission of Zdenek Herman.

**Fig. 15:**
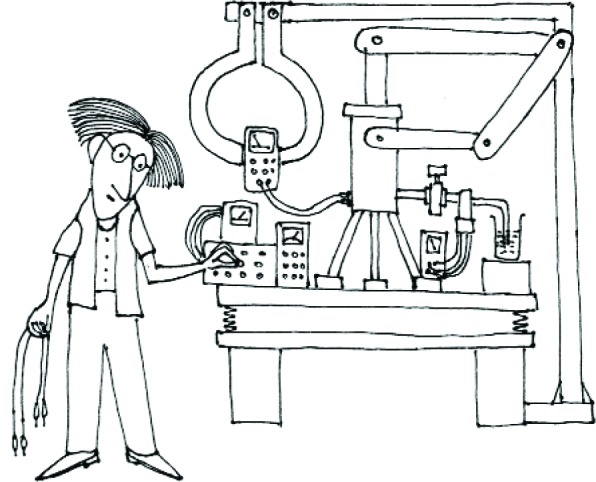
The power electrician. (Used with permission of Zdenek Herman.)

Additional implementing technologies included small vac-ion type pumps. The massive mechanical and diffusion pumps shown in Fig. 13 were no longer necessary to reach the required 10^−4^ Pa. Computers (Fig. 14) to deal with this fringe data stream were becoming increasingly faster while, at the same time, also getting smaller and smaller. The power requirements of electrical devices were also getting smaller (Fig. 15). (These three instrument-concept figures together with Fig. 7, the theorist’s view, were drawn for me by Zdenek Herman to illustrate the human tendency for “experts” to assume that their specialty will dominate a design. On the other hand, a good scientist needs to employ all the available “specialties” in just the proportions required for a particular project.)

**Fig. 16:**
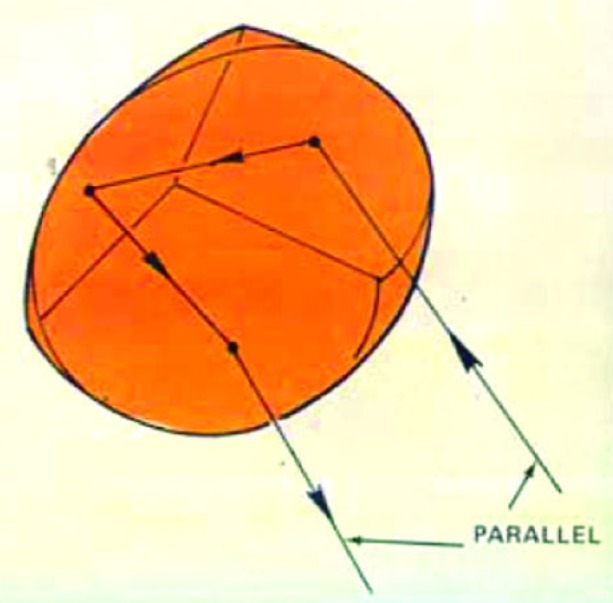
Retroreflector ray path.

Finally, retroreflectors had become recognized as useful optical devices, and corner cubes—the most convenient retroreflector type—were becoming commercially available. A retroreflector (Fig. 16) is an optical “mirror” that reflects an incident light beam back parallel to itself, *independent* of the orientation of the mirror.

**Fig. 17:**
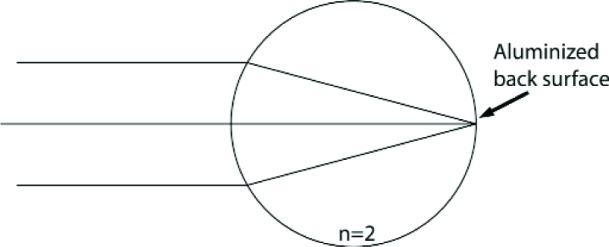
Glass sphere retroreflector.

**Fig. 18:**
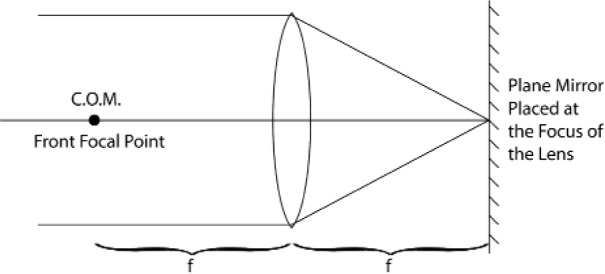
“Cat’s-eye” retroreflector.

Retroreflectors have an additional feature that is essential for their use in dynamic (moving) systems. They possess an “optical center” around which the inevitably occurring rotations will not affect the optical phase of the retroreflected beam [11]. For the simplest retroreflector, a glass sphere of optical index 2 (Fig. 17), the existence of an optical center is self-evident. For a “cat’s-eye” retroreflector (Fig. 18), it takes a moment’s thought—having been told the answer—to realize the fact that the front focal point of this lens-mirror combination provides such an optical center, i.e., rotations about this front focal point leave the phase of the retroreflected beam unchanged. The “optical center” for a cat’s-eye type of retroreflector–and the concomitant requirement that the center of mass of the dropped object be co-located there—forces one to work with a rather extended object.

An open corner cube consists of three mutually orthogonal mirrors; its optical center is at the common apex. For a solid corner cube, the optical center is the position of the apex as seen by an observer looking into the front face of the cube—a location that results in a compact dropped object. Locating the center of mass at the optical center with the requisite accuracy, however, involves both care and experimental cunning. For a free-fall rotation rate equal to the rotation rate of the minute hand of a watch, the center of mass must be placed within a few thousandths of a centimeter to keep the rotation-caused accelerations below a few parts in 10^9^ of the free-fall acceleration, *g*.

In the case of Artyom Vitouchkine’s and my most recent absolute gravimeter [12], a cam-based instrument that will be described later, this co-placement of the center of mass and optical center must be done even more precisely. Because the device makes 3.3 measurements each second and everything happens without any “time-outs,” inevitably somewhat larger rotations result. In our efforts to better co-place the optical center and center of mass, we made use of a well-known and quite useful Scientific Principle, namely “it’s always easier to make things worse.” To position the center of mass, we purposely “shook” the instrument so as to exacerbate the rotations that occurred. To effect the co-placement, we adjusted the position of the center of mass by moving an appropriate “tuning” mass until the answer obtained for *g* with “normal rotation rates” was the same as that obtained with exaggerated rotation rates. Using this criterion, we were able to locate the center of mass to better than a wavelength of light—far better than the 10^−5^ m uncertainty that can be obtained using mechanical measurements in combination with careful balancing [13].

## 3. Why Measure Little *g*?

**Fig. 19:**
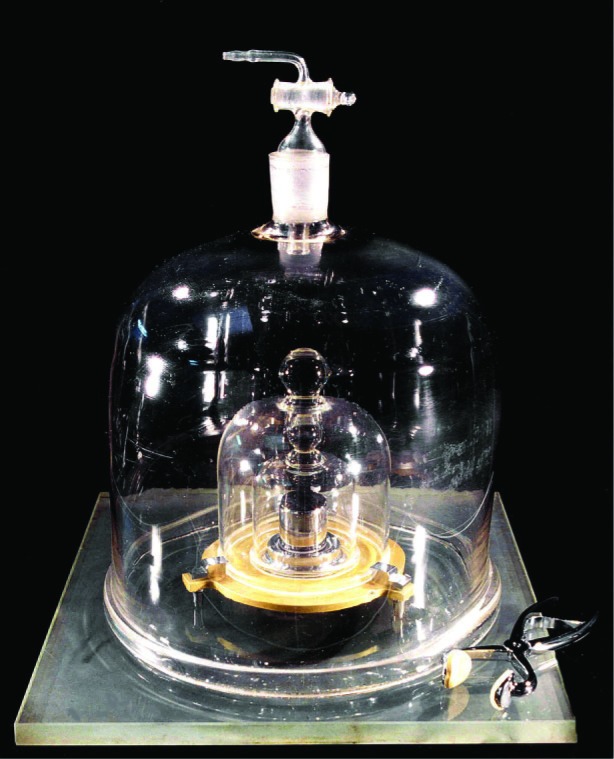
The kilogram. [Source: BIPM (International Bureau of Weights and Measures/Bureau International des Poids et Mesures, www.bipm.org).]

Certainly by this point the reader must be wondering: Why bother to measure *g*? And, why measure at the parts in 10^9^ level of uncertainty that is obtainable before the “sea of systematics” drowns the resultant accuracy? I offer two reasons. The first is the kilogram (Fig. 19), assuming it is not defined out of business [14]. However, even should this end up being the case, one would still want to connect the kilogram to electrical units via Watt-balance experiments. And, the transfer requirement to get a mass from an electrically balanced weight is knowing the absolute acceleration of the value of *g* at the measurement position of the Watt-balance [15].

**Fig. 20:**
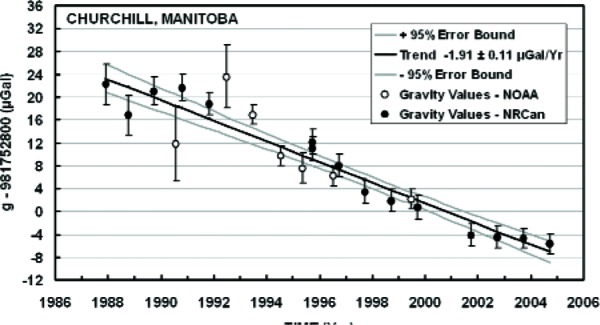
Churchill gravity. (Used with permission of Tony Lambert.)

**Fig. 21:**
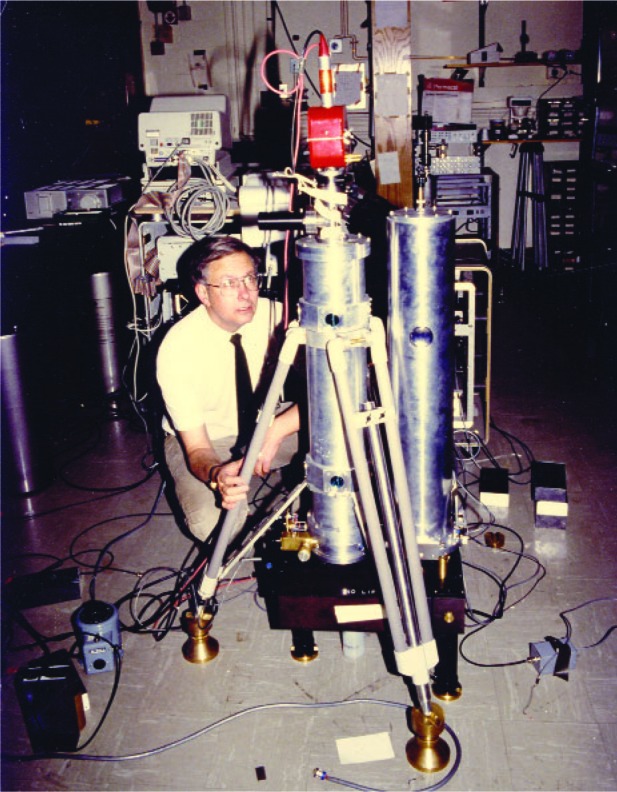
JILAg absolute gravimeter (lab photo).

The second reason can be found in a letter sent to me in 1982 from the University of Hannover’s Institüt für Theoretish Geodesie, now renamed the Institüt für Erdmessung, literally “Earth measurement.” This renaming correctly reflects the fact that during the past 20–30 years, the subject of geodesy has—as a result of advances in measurement technologies—been transformed from a chiefly theoretical endeavor that explored beautiful theorems, involving the Laplace and Poisson equations, to an experimental science—a science that has come of age as one can now make real-time measurements at the levels at which geophysical happenings are taking place. And absolute gravimetry (along with very long baseline interferometry, global positioning satellites, satellite ranging, etc.) was and is contributing to this revolution. As one example of this, consider the graph entitled “Churchill Gravity” (Fig. 20). It shows a monotonic downward drift in the measured value of *g* at Fort Churchill in Canada [16]. The observations are a direct measurement of post glacial rebound, which is still ongoing as the Earth’s crust is recovering from the last ice age! The first half of the Churchill data points were made using JILAg absolute gravimeters [17–19]. Built in response to requests from various gravity users that we provide them with our latest technology, the JILAg instruments combined the instrumental ideas that were developed [20] during the thesis work of Mark Zumberge [21] with the “super spring” idea [22] that was developed during the thesis work of Bob Rinker [23]. These particular scientific users chose not to reinvent the technology, which would not have been either wise (the instrumentation does have some subtleties) or cost effective (a rediscovered wheel still involves a lot of expensive new learning). Figure 21 shows the author (some time back) with one of the six JILAg absolute gravimeters, seen here in the JILA “spec-lab.” The latter half of the Fort Churchill measurements were made using FG5 commercial gravimeters [24], instruments that will be discussed later.

The JILAg instrument consists of a dropping chamber, seen in the center of Fig. 21; a “super” (isolating) spring, seen on the right; and the necessary timing and processing electronics, seen behind. During the construction phase of the six instruments, an opportunity presented itself to use the dropping chambers from two instruments to do “new science,” and in the course of doing this new science, as is often the case, an important lesson was learned.

## 4. Force Controversy

**Fig. 22:**
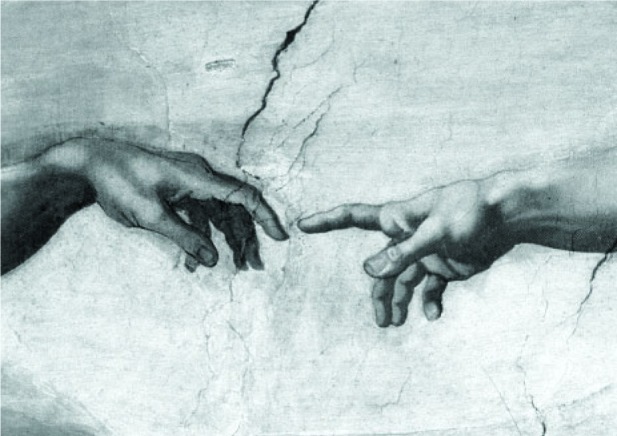
Sistine Chapel ceiling detail.

During the fall of 1986, the suggestion was made (based on a reanalysis of the early equivalence experiments of Baron von Eötvös) that gravity might have an up-to-this-point unrecognized short-range (centimeters to kilometers) component—dubbed the 5th Force [25]. Figure 22 offers the reader one example of a “short-range” type of interaction. The popular appeal of this suggestion (“gravity” is appreciated by all yet really understood by none) was evidenced on the front page of the next day’s New York Times under the caption “Hints of 5th Force in Universe Challenge Galileo’s Findings.”

**Fig. 23:**
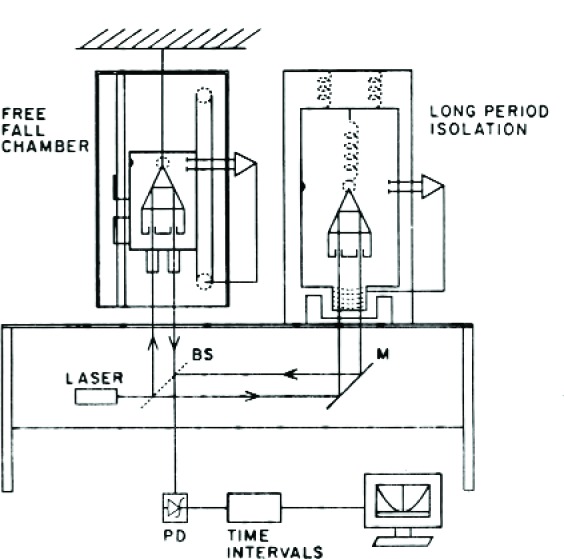
Schematic of JILAg gravimeter.

So what did we do? Naturally, we decided to see who was right, Newton or the 5th Force advocates. To understand what we did, one first needs to understand the working principles of the JILA gravimeter (Fig. 23). The free-fall chamber contains a “drag-free” motor-driven chamber that houses the one mirror of the interferometer: a corner-cube-containing dropped object. Initially, the chamber is driven down faster than *g* until a few millimeters of separation is achieved between the dropped object and its kinematic support in the chamber. Once this “lift-off” is achieved (and the dropped object is now in free fall), the motion of the chamber is forced to maintain a constant separation by servoing the drag-free chamber to track the motion of the freely falling object. At the bottom, the separation is servoed to a very small separation distance at which point the motor reverses the motion of the chamber with the result that the object is (gently) caught and returned to the top. After a small pause of between 2 s and 10 s (allowing various drop-induced variations to settle down), the entire measurement process is repeated. The “long-period isolation” chamber contains a “super spring”—a simple spring servoed to create, in effect, an effective kilometer-long spring with a period of ≈30 s. Hanging the reference “mirror” of the interferometer on such a spring serves to position it—as is the falling object–in vibration-free inertial space. Incidentally, vertical motions of the interferometer base produce equal changes in the lengths of both interferometer arms. Finally, a stabilized laser is used as the light source.

**Fig. 24:**
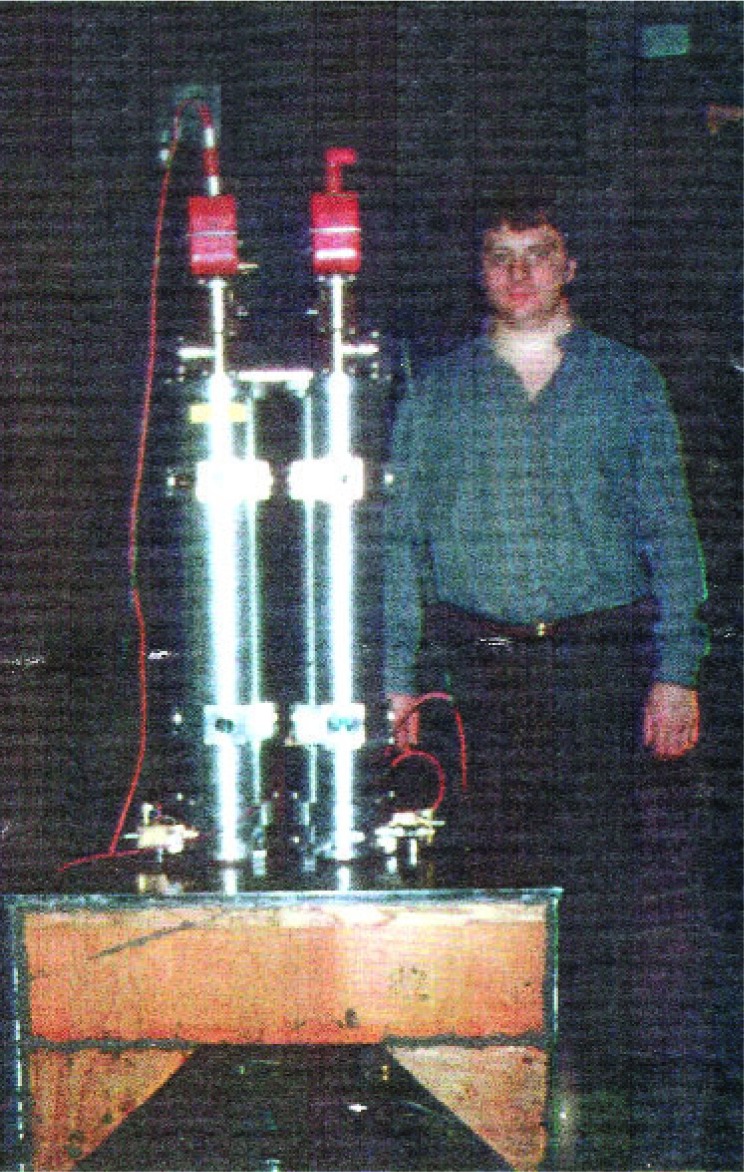
Galilean apparatus (lab photo).

**Fig. 25:**
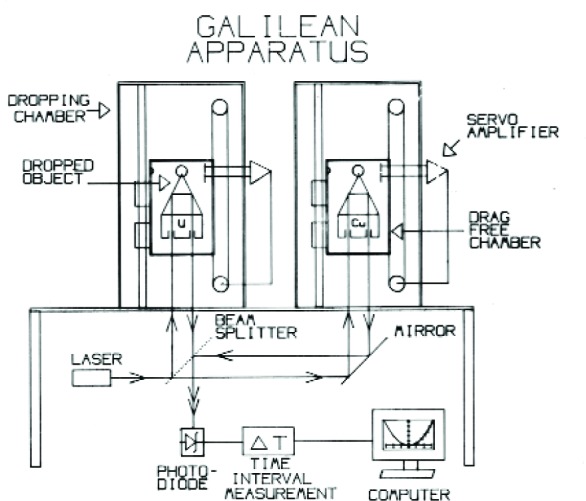
Schematic of Galilean apparatus.

The Galilean apparatus, with Tim Niebauer on the right (Fig. 24), is seen diagrammatically in Fig. 25. It consisted of two dropping chambers that sat on a wooden interferometer base. We chose wood because time was of the essence in either confirming or shooting down the 5th Force hypothesis. And actually, wood is a wonderful metrological material. It is good at damping out vibrations, and its along-grain temperature coefficient of linear expansion rivals invar!

We created a free-fall equivalence experiment, having “borrowed” two dropping chambers that were available during the process of constructing the six JILAg gravimeters, by adding a copper mass to one dropped object and a (depleted) uranium mass to the other. By releasing both “at the same time” we were able to reproduce the Leaning Tower of Pisa experiment that Galileo is given credit for but probably never actually performed. As a matter of experimental technique, rather than releasing the masses simultaneously, which would put the information regarding their possible different free-fall rates at a very low frequency, we released first one, then a moment later the second. In the case that they accelerate at the same rate, this technique yields a constant difference frequency of the order of 1 MHz for a few millimeters of free-fall separation. Our direct Galilean-type free-fall equivalence experiment [26] had an uncertainty of 5 × 10^−10^ and served to rule out the particular type and size of short-range interaction that had been suggested. Plus—and this was a BIG plus—we learned something!

The important lesson was that we did not do quite as well as we thought we would be able to do, given that it was a differential experiment in which most errors would be expected to cancel out. The explanation was that the release of two similar-weighing objects did not produce equal recoils from the less than uniformly rigid supporting floor, and this resulted in a floor (and subsequently an instrumental) tilt that preferentially shortened one interferometer arm more than the other.

Consequently, when the JILAg instrument was “technology transferred” to Axis Instruments which later evolved into Micro-g Solutions, the resulting FG5 instrument was—to minimize this tilt sensitivity—designed with the dropping chamber directly above the super spring. We should have thought of this earlier, but it took this “slightly poorer than it should have been” equivalence experiment to drive home the point that a straight-line instrument has a quadratic tilt dependence, whereas a side-by-side instrument has a tilt dependence that is first-order sensitive to the angle of tilt.

**Fig. 26:**
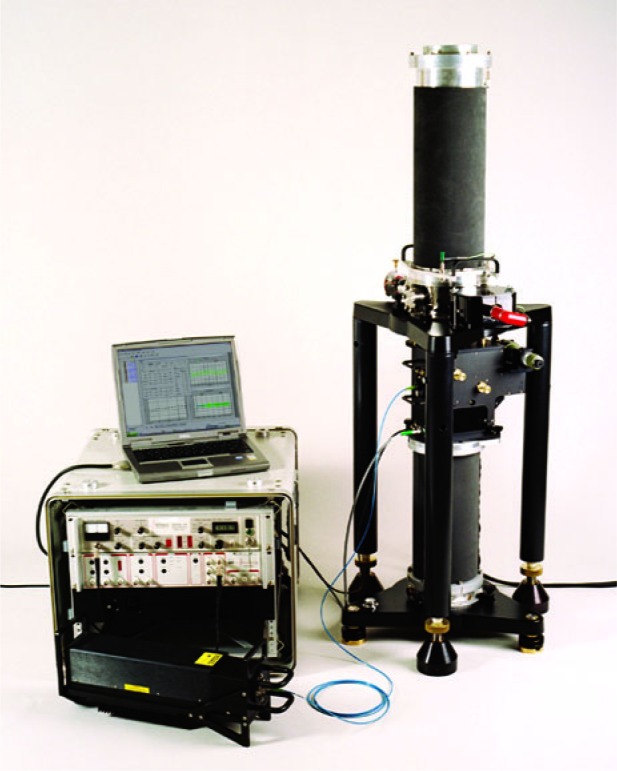
A commercial gravimeter. (Photo used with permission of Tim Niebauer, president Micro-g Solutions.)

As a result of this design change, the commercially manufactured absolute gravimeter now satisfied Abbe’s dictum: If you want to measure something in a particular direction, then build the apparatus along that direction and avoid measuring in orthogonal directions, which will likely introduce cross-couplings such as—in the case of the “side-by-side” JILAg gravimeter—a tilt sensitivity. Thus came about the commercial FG5 gravimeter shown in Fig. 26, of which, at this writing, more than 40 instruments have been sold for use in a variety of standards, gravimetric, and geophysical applications.

Another consequence of gravity having an unrecognized short-range component would be that the exponent in Newton’s law of gravitation (i.e., $F=G\frac{m_{1}m_{2}}{r^{2}}$) would not be exactly 2. This possibility had previously been investigated—prior to the 1986 5th Force suggestion—by Holding, Stacey and Tuck [27], who figuratively and literally worked “down under” in Australia. Their approach was to use spring-based relative gravimeters to measure gravity down a mine shaft. They recognized that short-range deviations from the inverse-square law would cause gravity to increase at a rate different than that calculated using 1/*r*^2^ measured down into the mine. And though this experiment proved more difficult than first imagined because of systematic errors arising from estimating the gravitational contributions from the less than homogeneous surface layers, it inspired a group at the Air Force Cambridge Research Center (following the 5th Force suggestion) to measure gravity up rather than down. They employed relative gravimeters at various heights on a television tower, and then compare their measured values with the values derived by upward-continuing surface gravity using an exact inverse-square relationship. Their result [28] seemed to confirm a breakdown at short ranges of the inverse-square law of gravitation.

In Erie, Colorado (20 miles from Boulder), there is a NOAA (National Oceanic and Atmospheric Administration) weather tower, which is 300 m tall. In light of the above result, I asked if we could use this tower to repeat (i.e., check) the Air Force tower experiment. NOAA asked a modest $1000 in rent for our use of the tower. Their other requirement was that we sign a paper to the effect that if we fell off in the course of making measurements, NOAA would not be held responsible for any personnel free falling due to gravity.

**Fig. 27:**
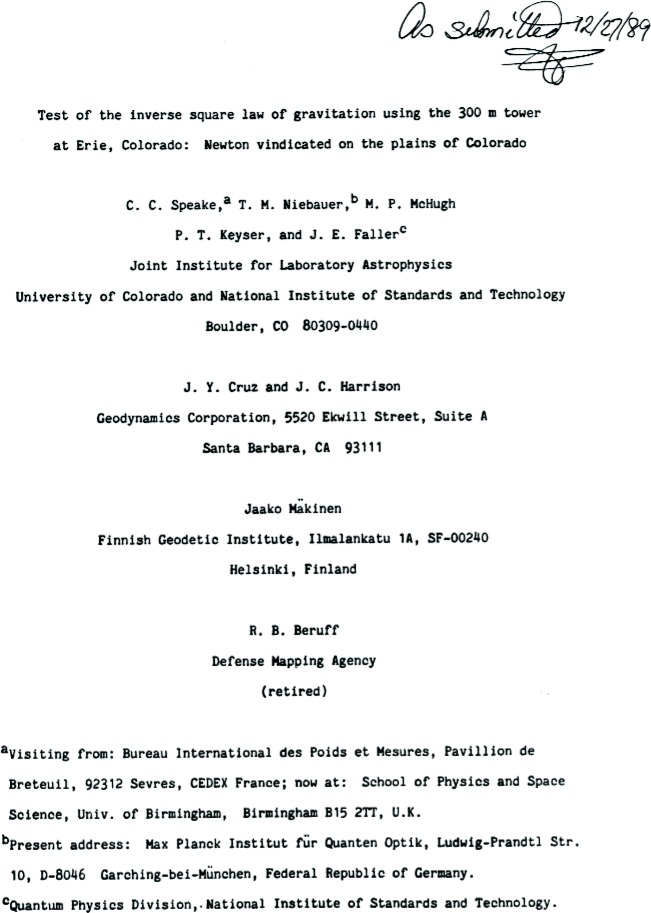
Our tower paper as originally submitted. The first version of this paper said, “Newton saved;” however, I had been talked into changing “saved” to “vindicated” prior to submission.

The result of our carefully executed experiment was that the exponent was found to be 2 within our experimental error. Figure 27 shows the first page of our paper as originally submitted. Unfortunately I could not “sell” its title to the editors of *Physical Review Letters* —apparently physics must be seen as a serious activity rather than as something that one might enjoy doing. The paper was accepted, however, with a more “appropriate” title [29]. In the end (numerous experiments by many workers later), Newtonian gravity was vindicated.

## 5. New Designs

**Fig. 28:**
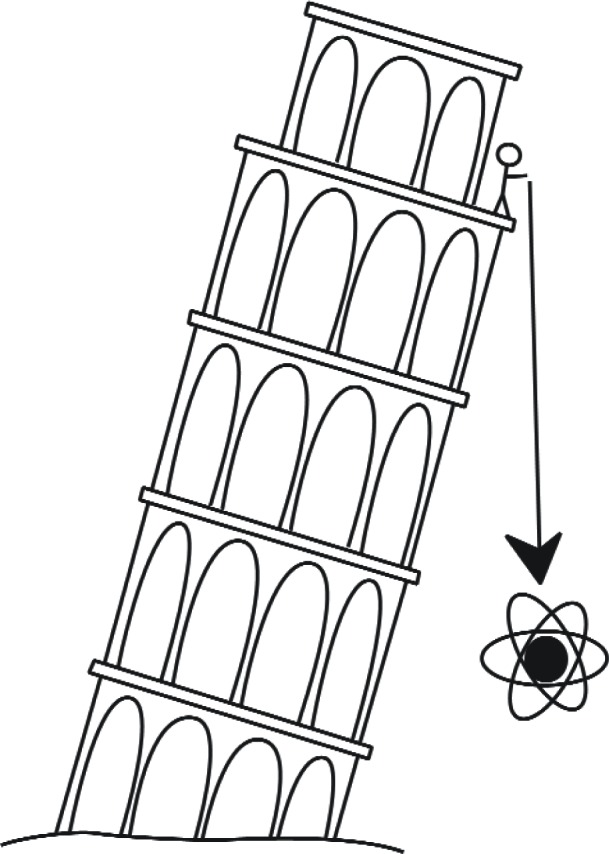
Dropping atoms.

What is next in the world of gravimetry? Perhaps future generations of absolute gravimeters will all measure *g* by dropping atoms (Fig. 28) [30]. Certainly today’s and tomorrow’s physicists are better trained to measure *g* by using microscopic atomic and molecular physics [30] than they are in dealing with the subtleties of “not-so-simple” macroscopic mechanical devices. However, it’s worth thinking about the gains to be had from dropping atoms. With atoms, there will be no recoil effects (atoms simply don’t weigh much), but certain critical parts of the rest of the apparatus still will need to be isolated. And, no isolating system, no matter how “super,” is perfect for isolating the needed macroscopic parts of the system, whether they be for an atom or for a corner-cube-based absolute gravimeter.

**Fig. 29:**
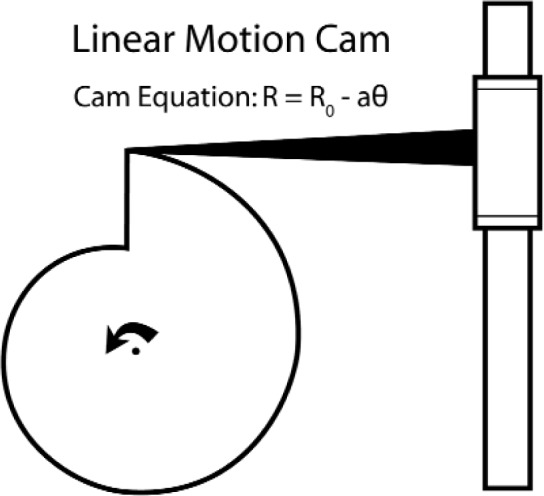
Linear motion-producing cam. The triangular cross bar riding on the cam causes the linear bearing to move down in a linear fashion.

**Fig. 30:**
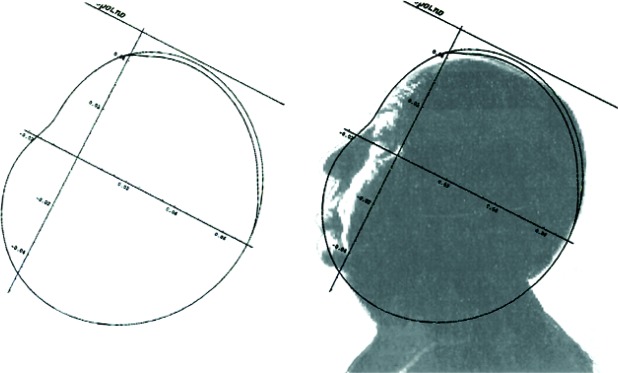
Free-fall cam shape. The outermost shape is that of the recoil-compensation lower cam; the inner shape is that of the upper cam which, in addition to creating the “drop” must also create the “lift-off” and “catch.” (The use of the profile is with the permission of the trustees of the Alfred Hitchcock Trust.)

Artyom Vitouchkine’s and my approach has been to develop a 6th generation macroscopic instrument, which uses a cam to create the repeated necessary motions of hold, release, catch, and lift. (This work served as the basis for Artyom’s Ph.D. thesis.) A cam of a type that produces a linear-in-rotation motion is shown in Fig. 29. Changing the cam’s shape to $\frac{1}{2}gt^{2}$ together with incorporating a smooth release, a gentle catch, and a quick return to the top, results in a considerably more complex cam—one, however, that is easily remembered (Fig. 30). Furthermore, and this is a critical “furthermore,” by using a second cam that drives an auxiliary mass, it is possible to have the center of mass of our free-fall system remain unchanged throughout the measurement cycle. Therefore (in principle) no recoil forces are created to act on the (always less than infinitely rigid) floor; and to the extent that this is perfect, we have achieved the “no-recoil advantage” that is to be had when dropping atoms.

Had I rushed to the library at some early point in the development of this cam-based absolute gravimeter, I might have come across—as I did later–Richard Phelan’s *Fundamentals of Mechanical Design* [31] in which he states, “With few exceptions, the most convenient means for imparting a specific motion to a member is by means of a cam-and-follower mechanism. Not only can the motion be completely specified, but the resulting physical configuration is both rugged and compact.” Oh well, so we rediscovered the wheel.

**Fig. 31:**
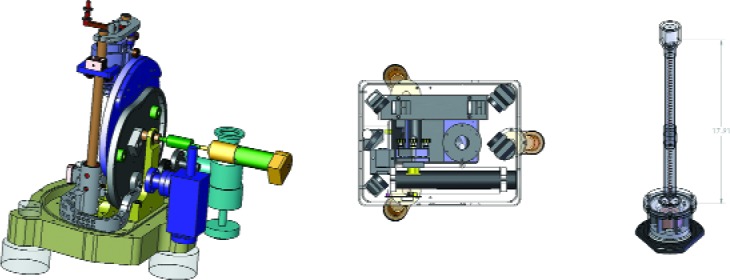
Design drawings: The cam drive, the interferometer base, and the isolating spring, which supports the reference mass.

**Fig. 32:**
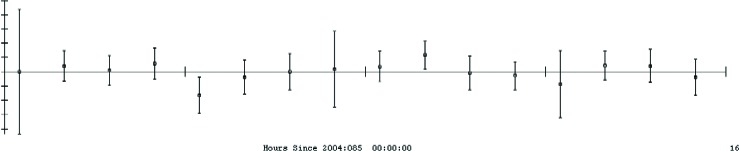
The proof of the pudding: Overnight run. The vertical scale ranges from +40 µg to −35 µg. The horizontal scale spans 16 h.

Figure 31 shows design drawings of the three mechanical parts of our new cam-based and recoil-compensated absolute gravimeter. This instrument makes 3.3 measurements per second with the result that it carries out 100 free falls in the course of 30 s! This instrument, which weighs less than 45 kg, has accuracy comparable to that of the larger FG5 instruments. At the same time, it is faster, lighter, and smaller—with the result that it will permit a wider use of absolute gravimetry both in field and laboratory applications. A typical data set from the new instrument is shown in Fig. 32. Each data point in this through-the-night run involves an average of just 30 seconds of data.

The fact that in this instrument we can use a “simple” spring to provide isolation (rather than an electronically based long-period super spring as required in the FG5 and JILAg gravimeters) not only serves to simplify the apparatus, but it also serves to remove any systematic effects due to electronic couplings between the dropping process and the spring’s electronics.

An examination of the data (Fig. 32) reveals that the average of each measurement set agrees with the other set better than expected for an $\frac{1}{\sqrt{N}}$ analysis. This occurs because the scatter is primarily due to the inevitable up-and-down motion of the spring-supported reference (corner-cube) mirror. However, since the period of the spring (1.2 s) was carefully chosen to be equal to four measurement cycles in length (something achievable as a result of our rapid measurement rate), the spring accelerations—though they cause scatter—do not shift the mean. The reason is that measurements spaced by a quarter of a wavelength along the spring’s high *Q* up-and-down motion average exactly to zero and therefore do not bias the result! At the present time, I am working on measuring the spring’s motion with an auxiliary optical position sensor and then applying electrostatic feedback to damp its motion to zero. Preliminary tests indicate that it is possible to damp the fundamental of the spring while having it still provide isolation for the reference corner cube at higher frequencies without introducing a systematic bias in the result!

**Fig. 33:**
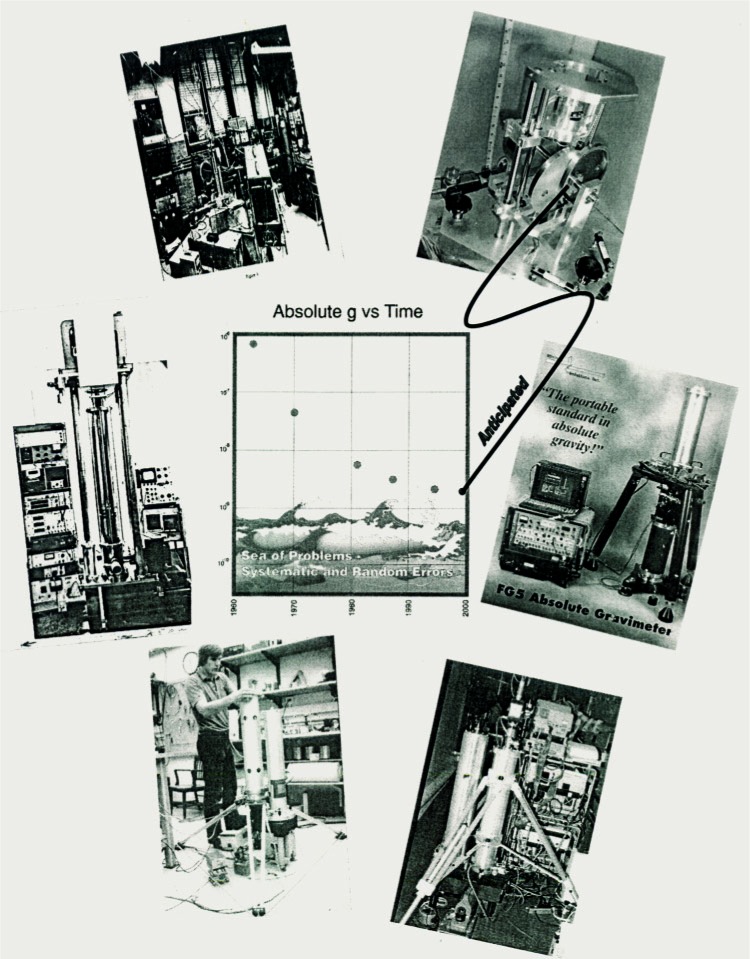
50 years of gravity instruments (lab photographs).

In summary, the montage of pictures in Fig. 33 shows the history of my involvement in the measurement of *g*. Happily, the accuracy of our new portable cam-type gravimeter appears comparable to that of an FG5. Its instrumental accuracy is indicated as the far right point in the center-of-montage curve of absolute *g* versus time.

## 6. To the Moon and Back

**Fig. 34:**
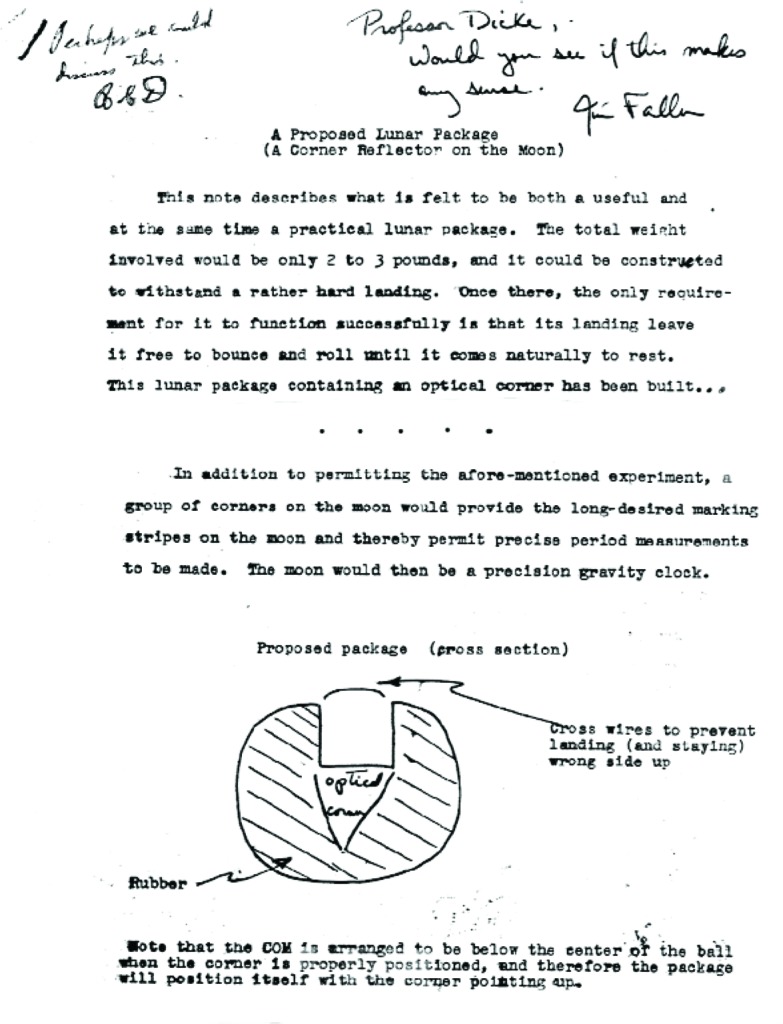
A proposed lunar retroreflector package.

**Fig. 35:**
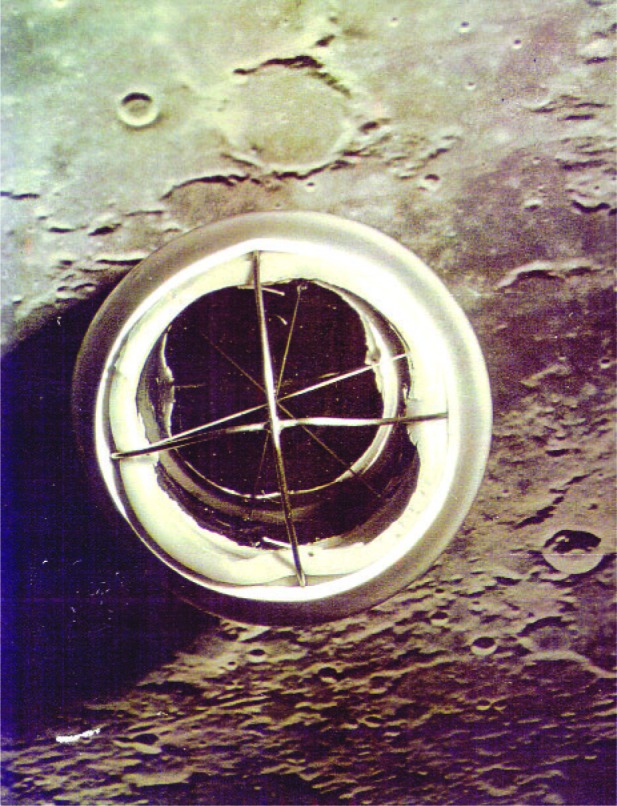
“Proposed” lunar package.

Before concluding, I would like to return to my secondary theme of how ideas (at least for experiments) come about. Let us go back to the late 1950s when I was learning about retroreflectors as rotation-friendly mirrors for use in optical systems. It was also during this time that a group from the Massachusetts Institute of Technology successfully received a photon echo from the lunar surface from a pulsed laser that was fired at the moon [32]. The echo’s returning pulse was inevitably spread out in time as a result of the sampled lunar topography. Putting this result together with my knowledge of the usefulness of corner cubes, I produced the “note” shown in Fig. 34 entitled “A Proposed Lunar Package (A Corner Reflector on the Moon).” My thesis advisor (R. H. Dicke) returned it annotated with the comment, “Perhaps we could discuss this.” The idea—for which I produced a model of a proposed lunar package (Fig. 35)—was to let one of the Surveyor missions roll onto the lunar surface a retroreflector mounted in a spherical container with its center of mass positioned such that the cube corner would come to rest facing up, thus providing the possibility of point-to-point, earth-to-moon (i.e., telescope-to-retroreflector) ranging.

**Fig. 36:**
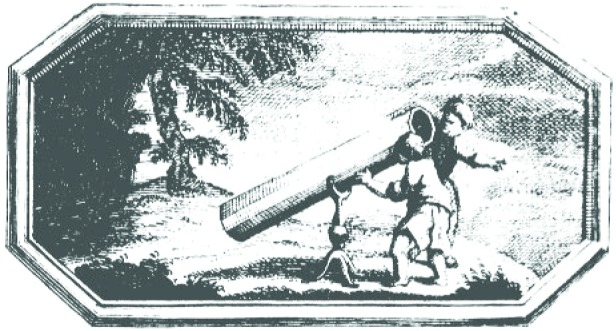
Lunar laser ranging: the idea. This figure appears on page 409 of Henry Pemberton’s book “A View of Sir Isaac Newton’s Philosophy” printed by S. Palmer, 1728, London. Figure information courtesy; of Special Collections, University of Colorado at Boulder Libraries.

**Fig. 37:**
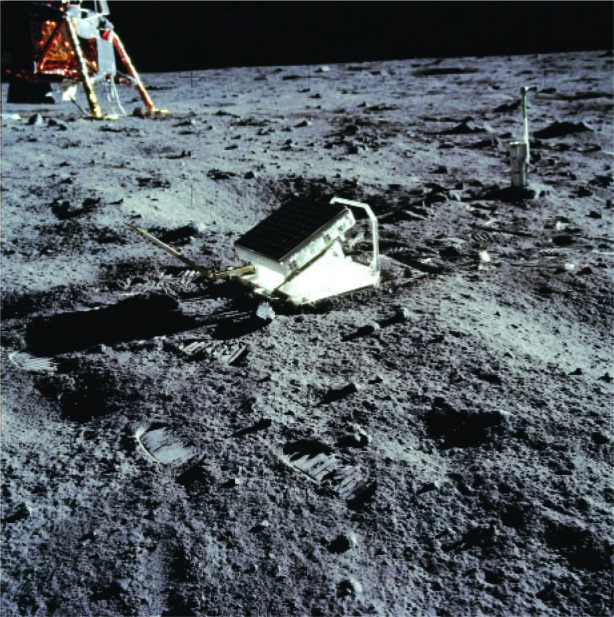
The Apollo 11 array on the moon.

The measurement idea was very simple (Fig. 36): A pulse of light is sent through a telescope to a retrore-flector on the lunar surface. Detecting the returned light pulse and accurately measuring its round trip travel time will then yield an extremely precise value for this earth-moon distance. From this relatively simple beginning evolved the lunar laser ranging experiment [33, 34] for which the Apollo 11, 14, and 15 astronauts placed arrays of retroreflectors on the lunar surface (Fig. 37). By observing the motions of the earth-moon (massive bodies) system over time, this “most-cost-effective” NASA experiment, amongst many other things, verified that gravitational self energy falls at the same rate as ordinary matter [35, 36]. Einstein, I think, would be pleased but not surprised.

**Fig. 38:**
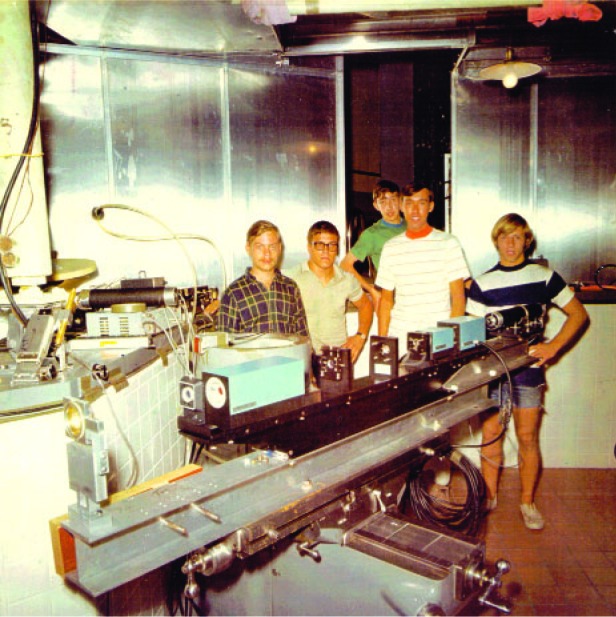
The “team” that first successfully ranged to the Apollo 11 reflector on the moon. From left to right are Steve Moody, Tuck Stebbins, Tom Giuffrida, Barry Turnrose, and Dick Plumb (lab photo).

To this day, the lunar arrays continue to be used for studies of gravity as well as the Earth and moon system—certainly the lunar laser ranging experiment is one of the triumphs of NASA’s Apollo program. Given the significance of this program, let me share a picture (Fig. 38) of the “team” that first ranged successfully to the Apollo 11 retroreflector array on the lunar surface shortly after that first visit by astronauts. As can be seen, they were not the mature scientists one might have expected, but rather they were a group of undergraduate students (from Wesleyan University in Connecticut) who joined me in bringing enthusiasm and the necessary skills to the task of detecting the first optical retroreflected returns from the Apollo 11 retroreflector array that was left on the lunar surface. Coincidentally, each of these young men now has a Ph.D. in physics.

## 7. Concluding Thoughts

**Fig. 39:**
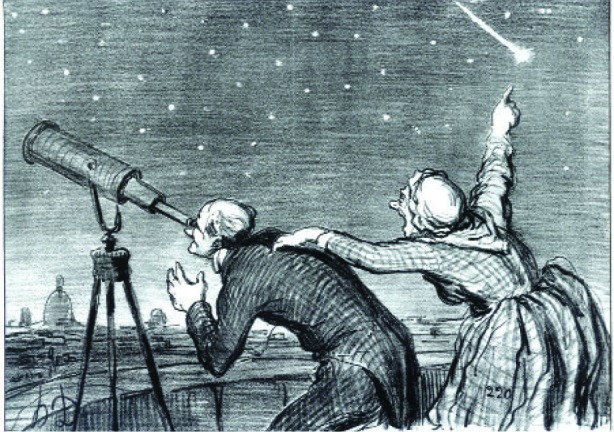
Remaining alert: Monsieur Babiner prèvenu par sa portiére de la visite de la comète (1858) par Honoré Daumier. (Photograph © 2005 Museum of Fine Arts, Boston.)

Let me now begin to conclude by making a few general remarks and observations about measurement science. Particularly in this field, where the pace is steady but necessarily slow, it is essential to remain alert to new scientific and measurement opportunities (Fig. 39). Metrologists need to be willing (and able) to redirect their research as new opportunities, measurement needs, and implementing technologies arise. À la Daumier, they need to maintain alertness to an ever evolving scientific sky.

In this article, I have shown the interconnectedness of precision measurement, namely how knowledge gained from making absolute measurements of *g* gave rise to two equivalence experiments—a direct differential free-fall experiment and a study of the free fall of massive bodies that showed that gravitational energy falls at the same rate as do other types of energy and mass. And equivalence lies at the heart of Einstein’s theory of gravitation.

**Fig. 40:**
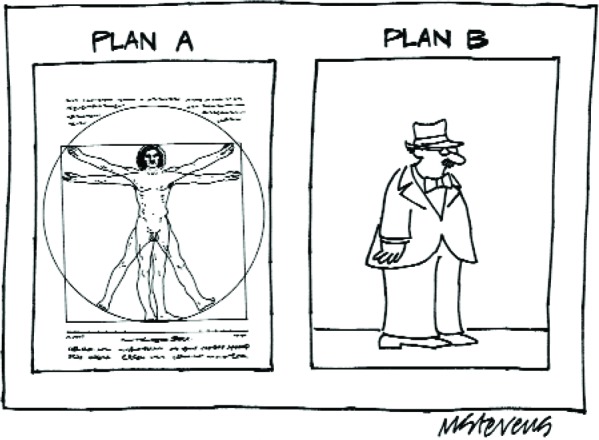
Theory versus experiment. (© The New Yorker Collection 2005 Mick Stevens from cartoonbank.com. All Rights Reserved.)

Clearly, and not just in his “1905 year,” ideas occurred to Einstein at a rate that can only cause the rest of us to stand in awe. Perhaps some solace is to be had in hearing about an ad entitled “Albert Einstein worked for us, will you?” that appeared in the January 16, 1998, issue of *Science* magazine. The ad stated that though Einstein submitted ideas to the Naval Surface Warfare Center (NSWC) for attaching mines to enemy ships, *none of his submissions was (deemed) feasible.* This may be a statement about the difference between plan A (read *theory*) and plan B (read *experiment*) (Fig. 40).

**Fig. 41:**
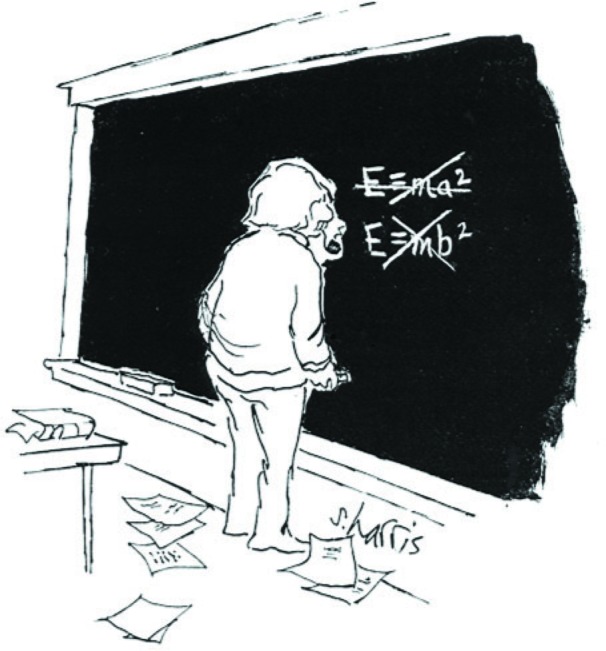
Einstein at the blackboard. (Used with permission of ScienceCartoonsPlus.com.)

**Fig. 42:**
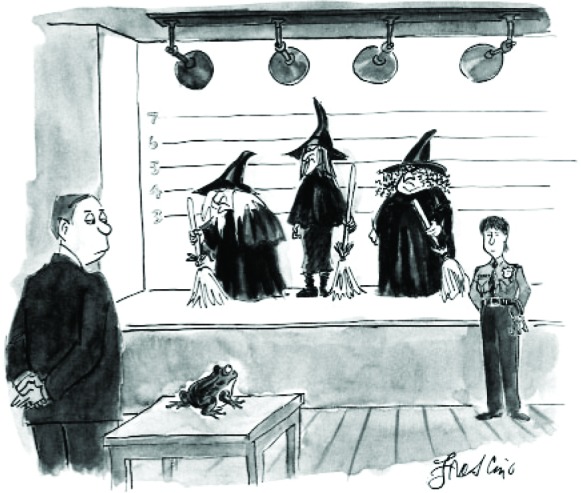
A former prince. (© The New Yorker Collection 1989 Ed Frascino from cartoonbank.com. All Rights Reserved.)

The field of precision measurement is a field of multiple and time-consuming subtleties—where “more things are known that are actually true” (attributed to J. R. Pierce). Vladimir Braginski (during a 2000 LISA meeting in Potsdam) said it this way: “To change the length of a wire is almost as difficult as changing (the) height of my wife.” Precision measurement science is also a field where the experimental physicist’s motto, “A month or two in the laboratory will save you an hour in the library,” should be carefully thought about. Equally applicable is the exhortation that appears on page 169 of the 1969 Alfa Romeo Shop Manual: “Never adjust more than one thing at a time or it will be impossible to tell which adjustment produced what result.” Precision measurement also requires an inordinate amount of patience, something which apparently Einstein had (Fig. 41). And, it is a field in which it is almost always difficult to recognize which source caused the problem (Fig. 42). Nevertheless, and in spite of the fact that it is a small and largely unrecognized field of science [37], precision measurement surely belongs in science’s “pedal line.”

**Fig. 43:**
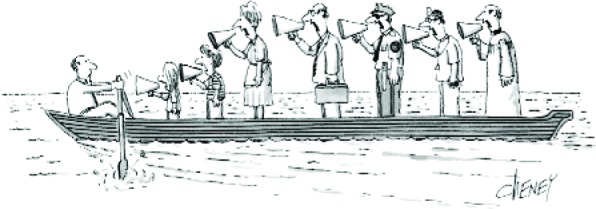
Calling the stroke. (© The New Yorker Collection 1983 Tom Cheney from cartoonbank.com. All Rights Reserved.)

**Fig. 44:**
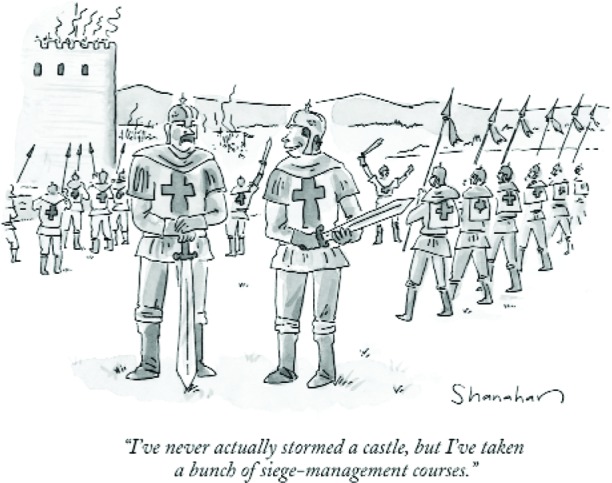
Management course graduate. (© The New Yorker Collection 2002 Danny Shanahan from cartoonbank.com. All Rights Reserved.)

**Fig. 45:**
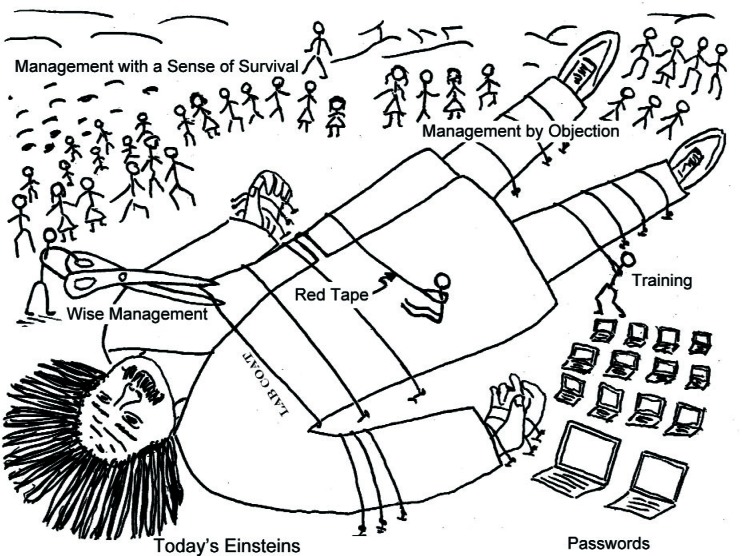
Gulliverian Constraints on Today’s Einsteins (Cartoon by J. Faller).

Given the importance of ideas like “suspenders,” how can one “manage” creativity? My suspicion is that in this case “less is more.” Certainly, having many “callers of the stroke” (Fig. 43) for each rower—no matter how well the callers have been trained in rowing management courses (Fig. 44)—is surely not the answer. My experience is that if you have hired creative and enthusiastic people in the first place, the best management involves little more than providing support (including the necessary infrastructure) and applause. As Einstein said, “It is almost a miracle that modern teaching methods have not yet entirely strangled the holy curiosity of inquiry; for what this delicate little plant needs more than anything, beside stimulation, is freedom [38].” The conventional (but unspoken) view of the scientist’s plight is shown in Fig. 45, “Gulliverian Constraints on Today’s Einsteins.” A wise management interested in encouraging creativity would be seen arriving with even more scissors.

Finally, let me conclude by suggesting what motivates and drives workers in this field. In *Nicholas Nickleby*, Dickens (in discussing Mrs. Nickleby, Chapter 43) says, “Pride is one of the seven deadly sins: but it cannot be the pride of a mother in her children, for that is a compound of two cardinal virtues—faith and hope.” This sentence, as I see it, contains the key ingredients for working in precision measurement: pride, faith, and hope.

Einstein said. “I have little patience with scientists who take a board of wood, look for its thinnest part, and drill a great number of holes where drilling is easy [39].” I hope you will take away from this article the recognition that precision measurement science is a thick board … as well as an important and fascinating area of science.

## Footnotes

1 Certain commercial equipment, instruments, or materials are identified in this paper to foster understanding. Such identification does not imply recommendation or endorsement by the National Institute of Standards and Technology, nor does it imply that the materials or equipment identified are necessarily the best available for the purpose.
